# Elucidating the role of WRKY27 in male sterility in Arabidopsis

**DOI:** 10.1080/15592324.2017.1363945

**Published:** 2017-09-18

**Authors:** M. Shahid Mukhtar, Xiaoyu Liu, Imre E. Somssich

**Affiliations:** a Department of Biology, University of Alabama at Birmingham, AL, USA; b Nutrition Obesity Research Center, University of Alabama at Birmingham, AL, USA; c Department of Plant Microbe Interactions, Max Planck Institute for Plant Breeding Research, Carl-von-Linné Weg 10, Koeln, Germany

**Keywords:** Anther dehiscence, plant biomass, pollen viability, stunted growth, Transcription factor

## Abstract

The WRKY proteins belong to a superfamily of TFs that play pivotal roles in responses to a wide range of biotic, abiotic, developmental and physiologic cues. Here, we assayed the accumulation of basal *WRKY27* transcripts in diverse tissue including root, shoot, leaf and flowers. We demonstrated that plants over-expressing *WRKY27* transcript levels exhibit growth aberrations and fertility defects. Scanning electron microscopic data suggest that *WRKY27* overexpressor plants exhibit pollen dehiscence defects. Our fluorescein diacetate hydrolysis assay showed that flowers of plants overexpressing *WRKY27* display significantly decreased pollen viability. These sterility-related phenotypes were not rescued by the exogenous applications of different phytohormones. Our results indicate the involvement of WRKY27 in particular for proper plant biomass accumulation and male fertility.

## Introduction

The regulation of transcription is a fundamental process in all living organisms and eukaryotes have evolved a complex mode of controlling expression of their genes. It is orchestrated by transcription factors (TFs) and other molecules working in concert to fine tune the levels of transcripts produced through a variety of mechanisms.^1,2^ TFs are essential components of the cellular machinery that regulate constitutively in some instances as well as modulate the spatiotemporal expression of downstream target genes in response to both internal and external stimuli.^1^ Coordinated transcriptional regulation ensures the precise functions of an organism. All aspects of transcription and its regulation involve dynamic interactions of TFs with chromatin; this fundamental property is known as an important modulator of biologic processes.^3,4^ Given that TFs are central to the regulation of gene expression, elucidating the molecular mechanisms of their actions is a major focus of research particularly since the complete genome sequence of *Arabidopsis thaliana* (hereafter Arabidopsis) became available in 2000.^5^

WRKY proteins are classified into one of the major families of TFs comprising 74 members in Arabidopsis.^6-8^ WRKY proteins contain one or two domains with a conserved peptide stretch of about 60 amino acids (termed WRKY domain) encompassing a novel zinc-finger motif. The WRKY domain shows a high binding affinity to a distinct *cis*-acting DNA element termed the W box (*TTGACC/T*).^6,9^ Research over the past 15 y demonstrated that WRKY factors are involved in regulating the expression patterns of a plethora of genes, by acting as positive or negative factors.^10-15^ Mounting evidence suggests that the WRKY proteins actively participate in a wide range of biologic processes. While the roles of numerous WRKY family members in plant biotic and abiotic stresses have been characterized,^3,11,15^ the underlying molecular mechanisms by which WRKY proteins participate in plant growth, development and flowering are not fully elucidated. Notable examples include WRKY10, also known as MINISEED3 (MINI3), and WRKY44, also referred to as TRANSPARENT TESTA GLABRA2 (TTG2) that play essential roles in seed and trichome development, respectively.^16-18^ Additionally, in Arabidopsis, WRKY71 was shown to accelerate flowering *via* direct activation of *FLOWERING LOCUS T* (*FT*) and the TF gene *LEAFY* (*LFY*),^19^ as well as affect shoot branching.^20^ In addition, WRKY6 and WRKY41 were demonstrated to be involved in seed germination and early seed development^21^ as well as in seed dormancy,^22^ respectively. Another pair of WRKY factors, WRKY2 and WRKY34, was shown to be required for male gametogenesis.^23^ Moreover, WRKY23 appears to control the maintenance of the root stem cell niche and to negatively regulate auxin transport.^24,25^ Finally, OsWRKY78 regulates stem elongation and seed development^26^ in rice. Here, we demonstrated the involvement of WRKY27 in male sterility. WRKY27, with a single WRKY domain within the C-terminus, belongs to group-IIe in the phylogenetic tree.^6,9^ WRKY22 and WRKY29 are the closest sequence homologs of WRKY27 that share 54% and 62% identity and 64% and 73% similarity, respectively.

## Results and discussions

**Figure 1.**
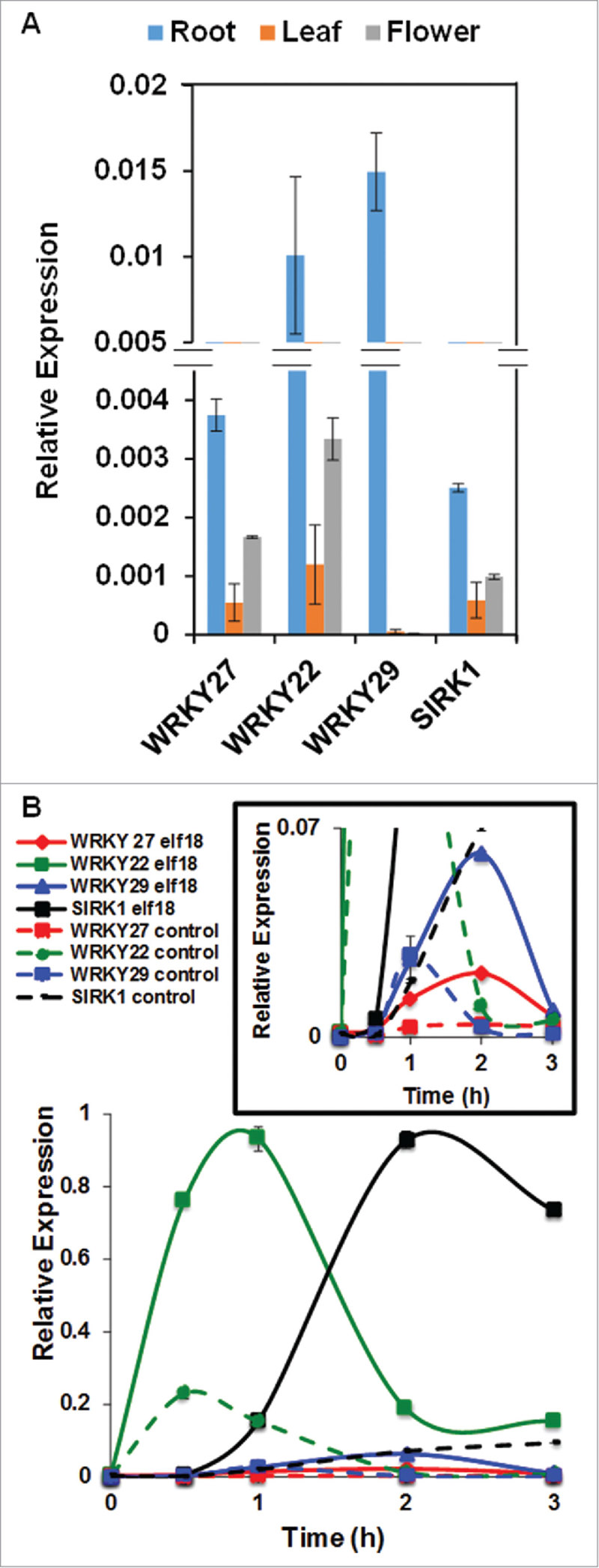
Measurement of *WRKY27* transcript levels in different tissue (A) Steady state expression levels of *WRKY22, WRKY29* and *WRKY27* in root, leaf, flower and silique-derived tissues were detected using quantitative real-time PCR. (B) Examination of *WRKY27, WRKY22, WRKY29* and *SIRK1* (Senescence-induced receptor-like kinase1) expression levels in leaf tissue upon elf18 treatment at indicated time points. The magnified view of expression data-related to *WRKY27* and *WRKY29* is shown (inset).

To decipher the potential functions of WRKY27 in plant development and reproduction as well as plant immune responses, we performed transcript analyses of *WRKY27* in diverse tissues and upon treatments with pathogens or pathogen-mimic stimuli. We also included WRKY22 and WRKY29, 2 WRKY family group IIe members most closely related to WRKY27,^6^ in our experiment to delineate the unique and overlapping expression patterns of these WRKY members under diverse physiologic conditions. Firstly, we performed a series of semi-quantitative and quantitative reverse transcription polymerase chain reaction (RT-PCRs) using mRNAs from root, shoot, leaf, flower and silique tissue in 21-day-old wild-type Col-0 plants. Overall, we detected relatively weak but differential transcript levels of *WRKY27* in the root, shoot, leaf and flower tissues (Fig. 1A; Supplementary Figure 1A and 1B). Intriguingly, none of the three tested WRKY family members showed expression in siliques under our experimental conditions (Supplementary Figure 1A). In our qRT-PCR analyses, we also detected relatively weak expression levels of *WRKY27* compared with *WRKY22* in all tissue types (Fig. 1A). In contrast, the expression levels of *WRKY29* were lower in leaf and flower compared with *WRKY27*. Moreover, we also detected relatively weak expression levels of senescence-induced receptor-like kinase 1 (*SIRK1*), also known as flagellin-induced receptor-like kinase 1, *FRK1*; a downstream transcriptional target of diverse WRKY members including WRKY22 and WRKY29^3,27,28^ in root, leaf and flower tissues (Fig. 1A). Our comparative organ-specific transcript analysis of *WRKY27* with other players is in concordance with the publically available transcriptomic data in The Bio-Analytic Resource (BAR) database^29^ (Supplementary Figure 1B). These data suggest common and unique functions of these three WRKY factors in plant development and reproduction. Finally, tissue-specific *WRKY27* expression patterns are supported by the GUS activity staining revealed in different parts of the plant including anthers and stigmatic papillae (Supplementary Figure 1C-1G) in our previously characterized *wrky27–1*, an exon trap mutant line, as well as in *P_WRKY27_:*GUS transgenic plants.^30^ These tissue-specific expression patterns of WRKY27 prompted us to consider the potential role of WRKY27 in plant reproduction, in particular anther development and pollen viability.

**Figure 2.**
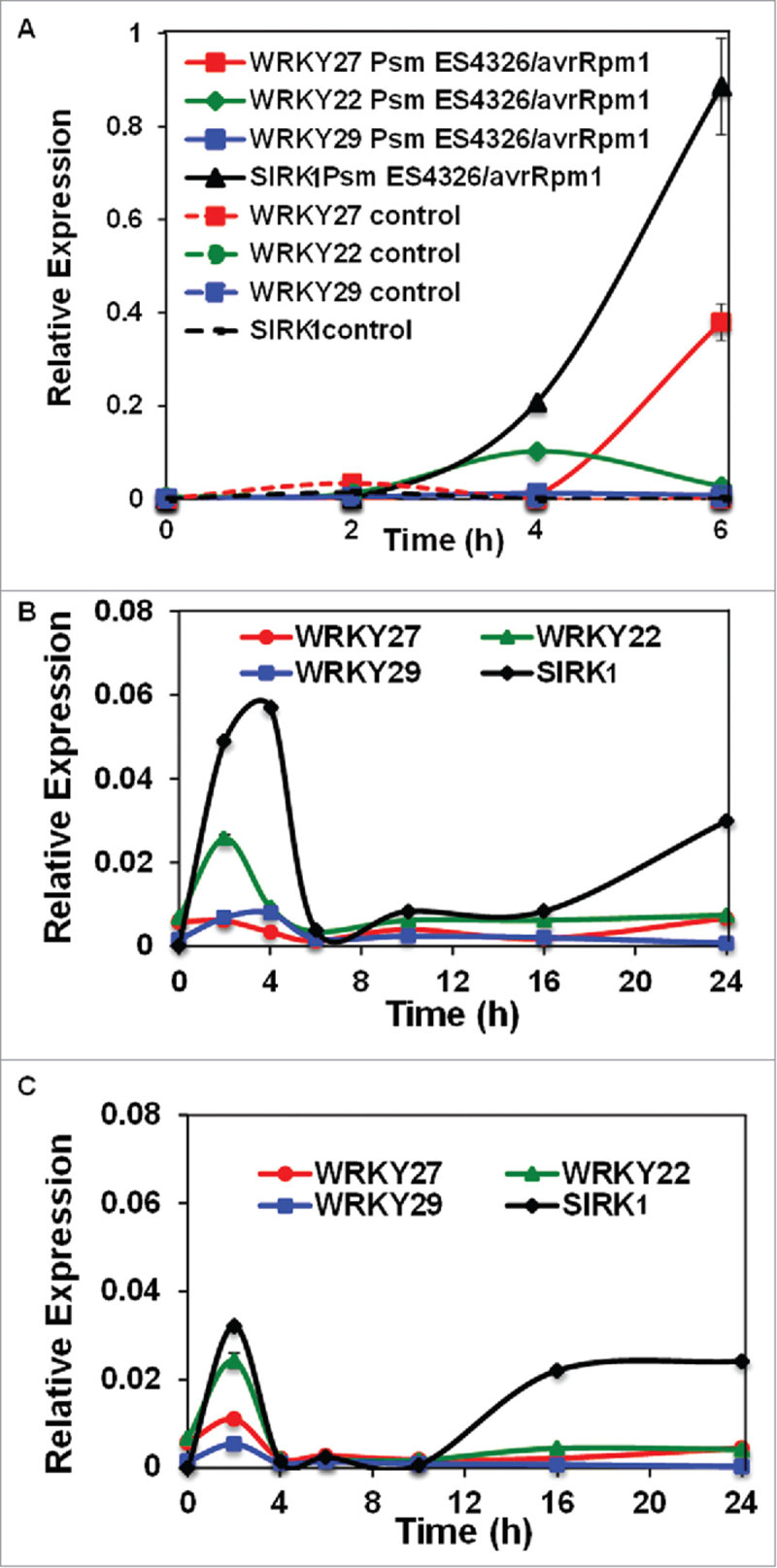
Detection of biotic stress-triggered expression patterns of *WRKY27, WRKY22, WRKY29* and *SIRK1* in leaf tissue. (A) mRNA levels of *WRKY27, WRKY22, WRKY29* and *SIRK1* detected using quantitative real-time PCR analysis in leaf tissue upon *Pseudomonas syringae* pv *maculicola* (Psm) ES4326 expressing avrRpm1 (*Psm* ES4326/avrRpm1) challenge. Accumulation of *WRKY27, WRKY22, WRKY29* and *SIRK1* transcripts in leaf tissue upon infection with *P. syringae* strain DC3000 (B) and *P. syringae* strain DC3118 (C), challenge. Time points after pathogen challenge are indicated.

In our previous study, we demonstrated that *wrky27–1*, which lacks a functional WRKY27 transcription factor, exhibited delayed symptoms development in response to the bacterial wilt pathogen *Ralstonia solanacearum*.^30^ These data corroborated with the basal and/or induced expression of *WRKY 27* in root and shoot vasculature. To investigate whether the transcript levels of *WRKY27* can be induced in the leaf, we performed an extensive qRT-PCR analysis using *WRKY27-*specific primers on cDNAs derived from leaf tissue subjected to a range of biotic stresses. This includes 10 µM elf18 (a peptide derived from the N-terminal region of bacterial elongation factor Tu), syringe-infiltration of avirulent strains of *Pseudomonas syringae* pv. *maculicola* (Psm) ES4326 expressing avrRpm1, spray inoculation of *P. syringae* strain DC3000 and *P. syringae* strain DC3118 (coronatine toxin-deficient mutant bacterial strain). We also included *WRKY22, WRKY29* and *SIRK1* as controls since these key immune players were demonstrated to be induced by several pathogens and pathogen-associated molecular patterns (PAMPs).^3,27,28,31,32^, As expected, we observed a strong induction of *WRKY22, WRKY29* and *SIRK1* at different time points upon treatment with elf18 (Fig. 1B). Intriguingly, we observed a moderate but significant induction of *WRKY27* at 1-hour, 2-hour and 3-hour post treatments with elf18 compared with mock at these specified time points. A similar observation was previously made in a publically available transcriptomics data set that used an unrelated PAMP, flagellin 22 (flg22; TAIR submission ME00332).^33^ Although *WRKY27* has a relatively low basal expression level, it can be strongly induced upon avirulent pathogen (Psm ES4326/avrRpm1) challenge, especially 6h post inoculation, akin to the expression pattern of *SIRK1* (Fig. 2A). Finally, we also detected various degrees of induction of *WRKY27, WRKY22, WRKY29* and *SIRK* upon infection with virulent pathogens DC3000 and DC3118 in our time course qRT-PCR assay (Fig. 2B, C). These data corroborate with the previously published microarray data that used DC3000 and DC3000-avrRpm1 for infections (TAIR submission ME00331).^33^ It's important to note that WRKY27, unlike WRKY22 and WRKY29, failed to induce *SIRK1* expression in transient assays^30^ suggesting the potential involvement of WRKY27 in PAMPs-triggered immunity as well as basal and effector-triggered immunity through a yet-to-be characterized mechanism.

**Figure 3.**
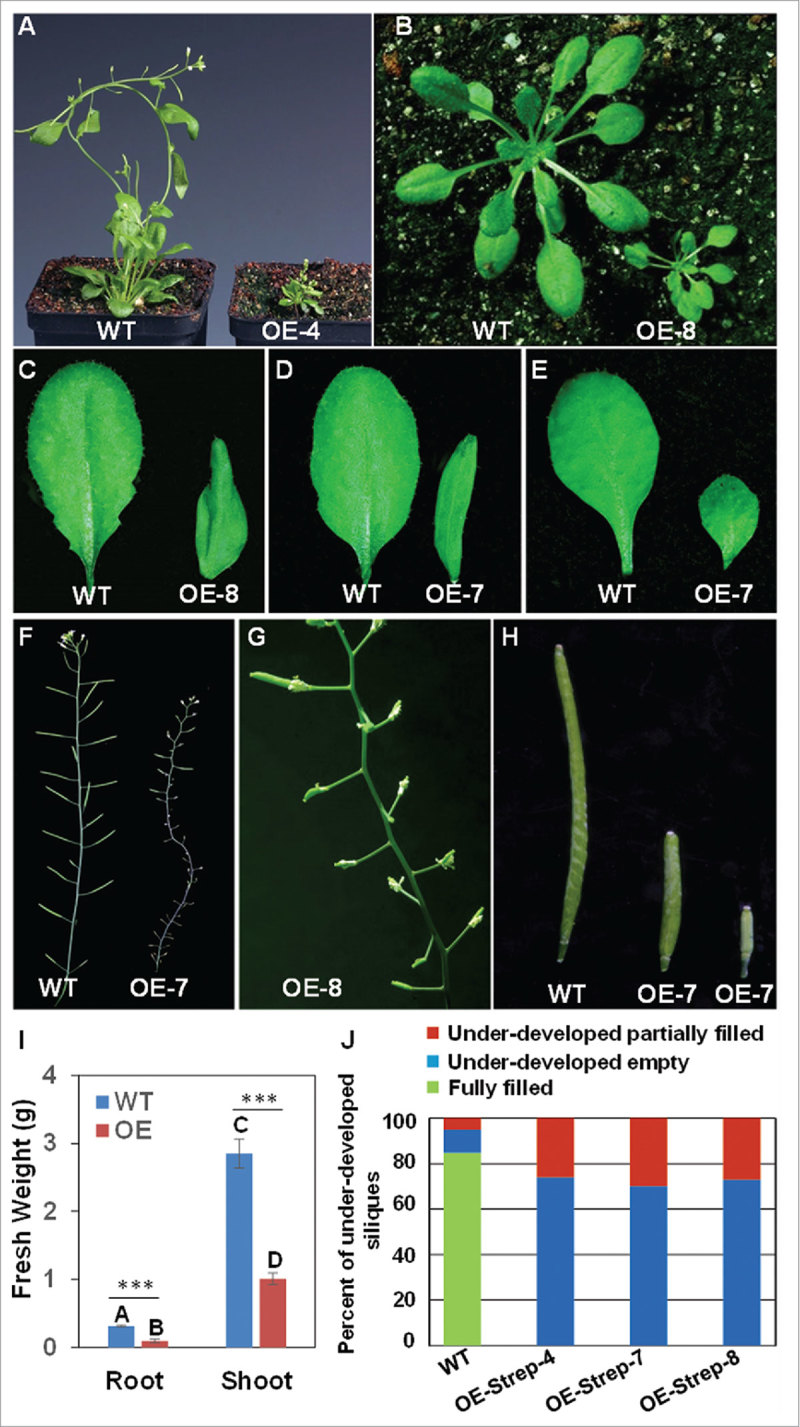
Pleiotropic phenotypes of ectopic *WRKY27* overexpressor (OE) plants. WT and OE denote wild-type plants and *WRKY27* transgenic overexpressor lines, respectively. (A and B) *WRKY27* overexpressor lines exhibit dwarf phenotypes compared with wild-type plants. (C–E) Altered leaf shape of *WRKY27* overexpressor plants compared with wild-type leaves. (F–H) Partial sterility and delayed senescence in perianth organs detected in plants expressing increased levels of WRKY27 compared with wild-type plants. (I) Root and shoot fresh weight of 5-week-old WT and OE soil grown Arabidopsis. Statistical analysis was performed by 2-way ANOVA followed by Bonferroni test, ***p < 0.001. (J) Quantification of under-developed empty and under-developed partially filled siliques.

While the roles of *WRKY27* in root and plant vasculature as well as the plant immune system were described previously,^30^ the expression of *WRKY27* in different floral tissues including anthers intrigued us to investigate its potential contributions in anther development and male sterility. At first glance, we did not observe any macroscopic floral-related phenotypes in the *wrky27–1* plants. This might be due to possible functional redundancy among members of the WRKY family. Potential candidates for such redundancy include the closely-related WRKY22 TF but also WRKY2 and WRKY34 that were previously shown to be required for pollen development,^23^ as well as WRKY31, WRKY42 and WRKY47 that showed elevated expression in pollen and stamen based on microarray studies.^29^ Such functional redundancy among TFs and other proteins has been well documented in Arabidopsis.^34,35^ For instance, plants lacking functional MYC5 TF exhibited no visible phenotypes, while overexpression of *MYC5* fused with a repressive domain showed male sterility.^35^ Thus, overexpression or missexpression of a gene product can provide an alternative and complementary approach to help define the functions of genes that could not be revealed using classical reverse genetics approaches.^36^ Hence, we generated a series of *WRKY27* overexpressing constructs using the full-length cDNA of *WRKY27* fused with the 3´ terminator element or in frame to a *StrepII* sequence allowing epitope tagging of the respective protein. We transformed the resultant constructs, *2 × 35S::WRKY27*-terminator and *2 × 35S::WRKY27-StrepII*-terminator into Arabidopsis Col-0 wild-type and *wrky27–1* mutant plants using *Agrobacterium*-mediated transformation.^37,38^ Northern blot and qRT-PCR analyses confirmed the overexpression of WRKY27 in several transgenic lines (Supplementary Figure 2A and 2B). We subjected these transgenic overexpressor (OE) lines for further comprehensive phenotypic analyses to elucidate the roles of WRKY27 in vegetative and reproductive phases of development. Compared with wild-type Col-0, plants overexpressing *WRKY27*, regardless of the transgene construct type (with or without an epitope tag), exhibited visible phenotypes associated with stunted growth during the entire vegetative growth phase resulting in a smaller plant stature (Fig. 3A, B). In addition, we observed that the leaves of *WRKY27* overexpressing plants were smaller in area and more curled when compared with corresponding leaves of wild-type Col-0 plants 4 weeks post germination (Fig. 3C-E). We also measured root and shoot biomass and demonstrated that (*OE)-Strep-4, OE-Strep-7* and *OE-Strep-8* transgenic lines display significantly reduced biomass in both above- and below-ground plant tissues compared with Col-0 (Fig. 3I). The most evident observation, however, was partial sterility and a delay in senescence in perianth organs. The young siliques of the overexpressor transgenic plants were underdeveloped and empty, and started to eventually elongate at very late stages of flowering (Fig. 3F-H). Subsequently, we quantified the number of underdeveloped empty and underdeveloped partially filled siliques in *OE-Strep-4, OE-Strep-7* and *OE-Strep-8* transgenic lines. We determined that over 70% of the siliques were underdeveloped and empty for these three representative *WRKY27* overexpressing plants, while less than 30% were partially filled (Fig. 3J). In any case, normal silique size was never attained and the seed content was significantly reduced, compared with those of wild-type plants (Fig. 3).

Given that concerted actions of plant hormones such as jasmonic acid (JA) and gibberellic acid (GA) are essential for proper stamen development^39^ and exogenous applications of these hormones or hormone mimics have been shown to rescue sterility-related phenotypes in Arabidopsis,^40,41^ we subjected the flowers of *WRKY27* overexpressor lines to phytohormone treatments. However, the sterility phenotypes of plants overexpressing *WRKY27* were not reversible by exogenous application of JA, GA and gamma-amino butyric acid (GABA).

**Figure 4.**
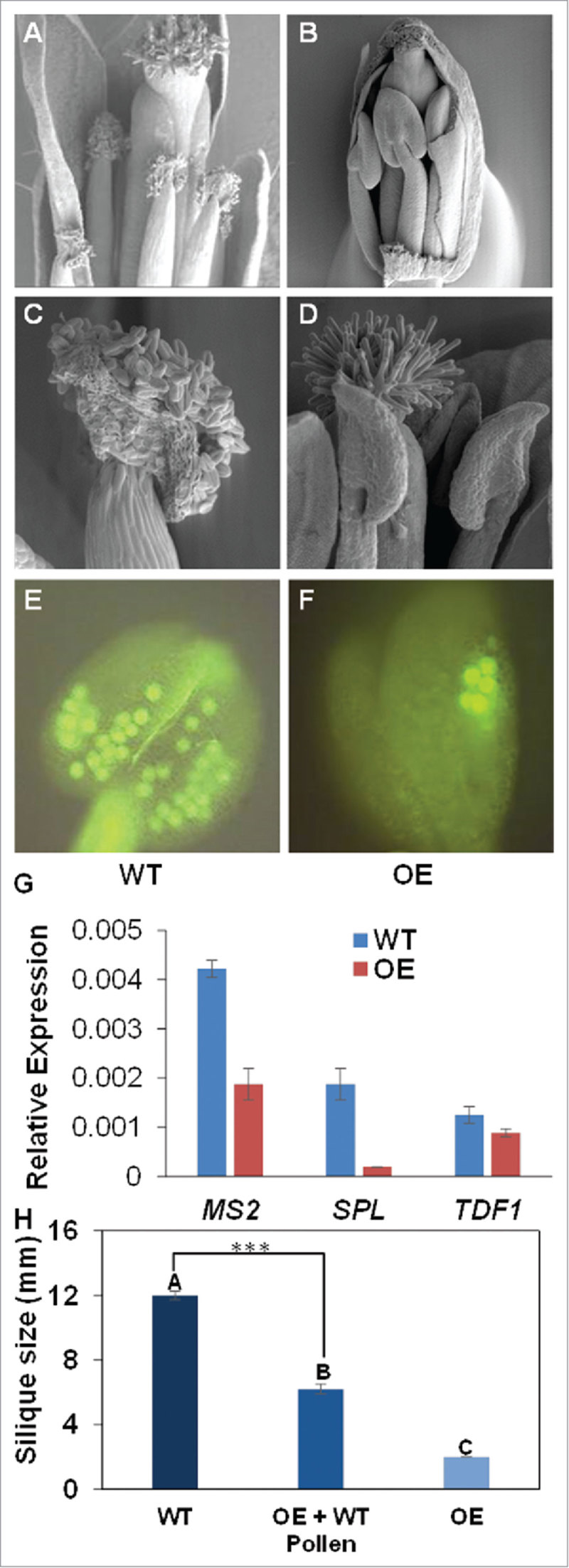
*WRKY27* overexpressor lines show reduced fertility. (A–D) Scanning electron microscopy (SEM) analysis of *2 × 35S::WRKY27* flower development. Unopened anthers of *WRKY27* transgenic overexpressor plants compared with those of wild-type plants. (E and F) Fluoresceine diacetate (FDA) assay on unopened anthers from *2 × 35S::WRKY27* overexpressor lines showing reduced pollen viability compared with wild-type plants. (G) mRNA levels of *SPL/NZZ* (SPOROCYTELESS/NOZZLE), *TDF1* (tapetal development and function 1) and *MS2* (male sterility 2) detected using quantitative real-time Polymerase Chain Reaction (PCR) analysis in floral tissue. (H) Quantification of silique length 7 d after pollination with WT pollens. Self-pollinated OE siliques were used as controls. Statistical analysis was performed by one-way ANOVA, ***p<0.001.

To better understand the nature of the sterility-related phenotypes, we investigated the floral structure of the *WRKY27* overexpressor plants in more detail using scanning electron microscopy (SEM). While anther dehiscence in the wild-type flowers normally occurs at the end of flower development, we observed severely delayed or completely inhibited anther dehiscence in the developing flowers of *WRKY27* overexpressor plants (Fig. 4A-D). These results indicate that the low fertility of plants expressing *WRKY27* might be caused by interrupted anther dehiscence. To search for defects in pollen quality, the pollen grains of *WRKY27* overexpressor lines were subjected to fluorescein diacetate (FDA)-based fluorochromatic reaction. This test evaluates the integrity of the plasma membrane of the pollen grains and is an easy, quick and accurate assay to monitor for viability of pollen grains in many plants, as only viable pollen will fluoresce under the microscope.^42^ Pollen grains from 5 independent *2xCaMV35S::WRKY27* overexpressor lines were stained with FDA. The count of fluorescent pollen grains revealed a significantly lower percentage of viable pollen derived from the overexpressor lines compared with the wild-type (Fig. 4E, F). ^43^

To corroborate our cellular studies, we examined the transcript levels of several well-defined major genetic components of anther and pollen development. This includes SPL/NZZ (SPOROCYTELESS/NOZZLE) that is involved in regulation of anther cell differentiation),^44^ TDF1 (tapetal development and function 1)^45^ and MS2 (male sterility 2) ^46^ that are involved at various phases of pollen and anther development. We demonstrated that *WRKY27* overexpressor plants accumulate lower levels of *MS2, SPL* and *TDF1* mRNAs,^43^ which may explain the deficits in male fertility (Fig. 4G). Finally, we artificially pollinated *WRKY27* overexpressor flowers with wild-type pollen at an early flowering stage, which resulted in partial restoration of fertility (Fig. 4H). Taken together, we concluded that overexpression of *WRKY27* results in male sterility.

## Conclusions

Overall, we discovered that *WRKY27* is expressed in diverse tissues including root, shoot, leaf and floral organs. While the requirement of functional WRKY27 in root and leaf vasculature was previously demonstrated, we expanded the potential roles of this important member of the WRKY family in leaf-centered immune responses. This finding will form the basis for future research. In the current manuscript, we focused to decipher the involvement of WRKY27 in floral organs and provide evidence of the dual functions of this key gene in plant defense as well as plant development and male sterility. We showed that the endogenous expression patterns of *WRKY27* overlap with the plant organs displaying the aberrant phenotypes. Finally, we also demonstrated that the transgenic plants with increased levels of *WRKY27* mRNA displayed several alterations in the morphological phenotype, related to stunted growth, irregular leaf shape, and drastically reduced male fertility. In summary, our results indicate dual roles of WRKY27 in plant immunity and development, in particular proper plant biomass accumulation and male fertility.

## Materials and methods

### Plant treatments, RNA and quantitative Real Time PCR (qRT-PCR)

Leaf tissue was collected from 4-week old Arabidopsis plants that were syringe-infiltrated with 10 µM flg22 (Genscript) or 10 µM elf18 (Genscript).

Pathogen treatments were performed using 4-week old plants that were syringe-infiltration with *Psm* ES4326/avrRpm1 (OD_600nm_ = 0.1), spray inoculated with *Pst* DC3000 (OD_600nm_ = 0.2, 0.02% Silwet L-77) or *Pst* DC3118 (OD_600nm_ = 0.2, 0.02% Silwet L-77). Untreated root, shoot leaf, shoot, and flower tissues were collected to determine the basal levels of tarnscripts. We extracted total RNA from the collected samples using RiboZol (AMRESCO). Possible genomic DNA contamination was eliminated using DNase I (Ambion). Formaldehyde agarose gel preparation, quantification, electrophoresis and samples prepration/loading were done following the RNeasy Plant Mini® Kit QIAGEN protocol. SuperScript III first-strand RT-PCR kit (Invitrogen) was used to convert mRNA into cDNA. We used GoTaq qPCR Master Mix (Promega) to perform qRT-PCR using gene-specific primers in a RealPlex S MasterCycler (Eppendorf). Primers used in this study are listed in the Supplementary Table 1.

### RNA hybridization and Northern blot analysis

Pre-hybridization and hybridization were performed in hybridization solution in glass tubes (30 cm × 4 cm) at 65°C under continuous rotation in a hybridization oven (Bachofer, Reutlingen, Germany). The pre-hybridization was performed overnight. Upon adding the denatured radio-active probe, the hybridization was performed for at least 16 hours. After hybridization the filter was washed with SSC and SDS solutions. The filter was wrapped in Saran wrap and exposed overnight to a phosphoimager screen (Molecular Dynamics) in a cassette at room temperature. 50–100 ng of gel-purified PCR product for Northern analysis was used. Probe was prepared according to Rediprime II Random Prime Labeling System protocol manual provided by Amersham Biosciences. Probe was later on purified on a Sephadex G25 column.

### GUS and FDA staining assays

Plant tissue were stained for GUS activity using a solution containing 2 mM 5-bromo-4-chloro-3-indolyl glucuronide (X-Gluc) in 0.1 M Na2HPO4, pH 7.0, 10 mM EDTA, 0.5 mM potassium ferricyanide/ferrocyanide, and 0.06% Triton X-100 at 37°C for 16 hours. The samples were cleared of chlorophyll by sequential washing in 70% ethanol. The fluorochromatic reaction (FCR) procedure was used for determining the viability of pollen. 0.02 g of fluorescein diacetate (FDA) (Sigma-Aldrich GmbH, Munich, Germany), was mixed with 10 ml of acetone. The FDA solution was added drop by drop to 5 ml of 20%sucrose until persistent turbidity was achieved. This solution was used within 30 min of preparation. Each slide containing 10 µl of this solution and pollens were incubated at room temperature for 15 minutes. Viability of pollen grains was examined under a fluorescence **microscope** (Leica MZ12, excitation filter 450–490 nm).

### Phytohormone treatments, biomass and cross-pollination

Diverse concentrations (1 mM to 0.5 M) of Gamma-aminobutyric acid (GABA) soluble in dH_2_O, 100 µM of Methyl Jasmonate (MeJA) and mock solution 0.2% ethanol,  1 µM GA_3_ solution (stock solution of 10 mM GA_3_) were sprayed directly on flowers. Rosettes of individual 5-week-old Arabidopsis plants were removed and weighed as shoot fresh biomass. Root of individual 5-week-old Arabidopsis plants were rinsed in water to remove soil before fresh biomass determination. To achieve cross-pollination, emasculation was performed one day before artificial pollination using pollens from designated plants. Siliques length was measured 7 d after cross-pollination.

## Supplementary Material

suppl_mat_Elucidating_the_role_of_WRKY27.pdf
