# Supplementary material for: Elucidating the role of WRKY27 in male sterility in Arabidopsis
Source: Plant Signal Behav. 2017 Sep 18;13(11):e1363945. doi: 10.1080/15592324.2017.1363945 (PMC5640203; doi:10.1080/15592324.2017.1363945)
Supplement: suppl_mat_Elucidating_the_role_of_WRKY27.pdf [file kpsb-13-11-1363945-s001.pdf]

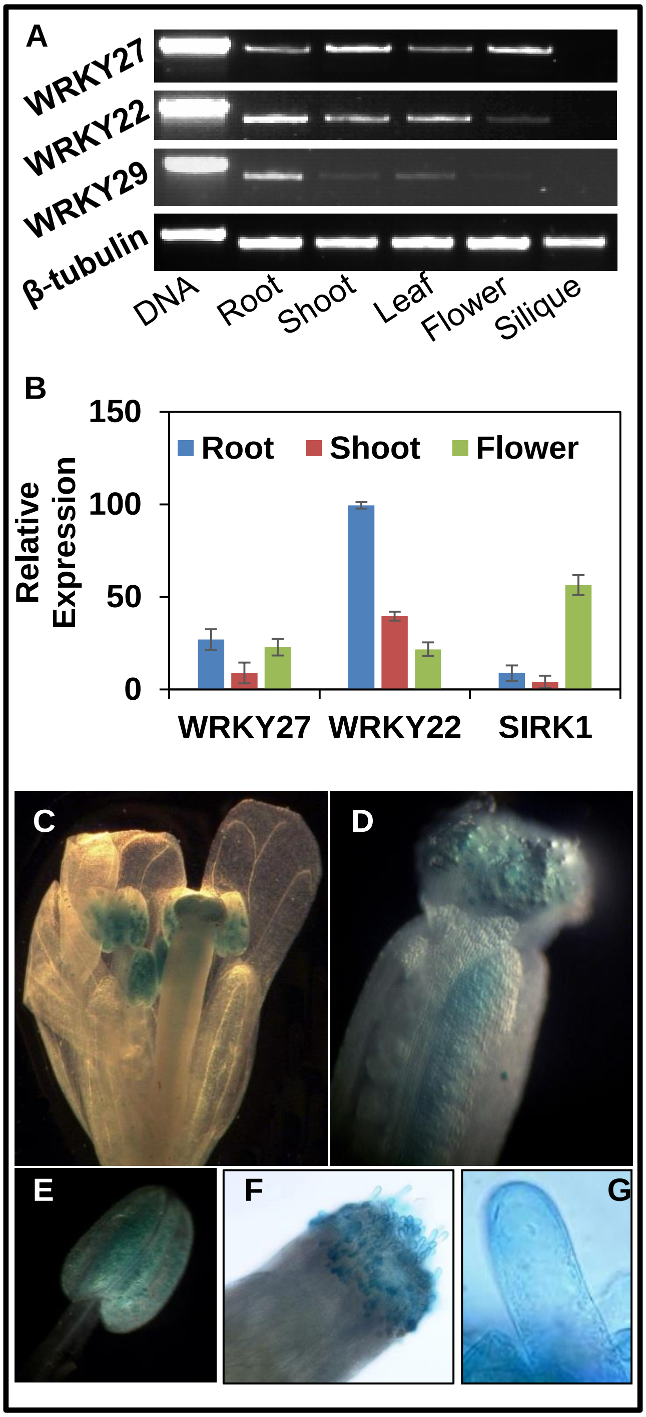

Supplementary Figure 1. Detection of *WRKY27* transcript levels in different plant tissues. **(A)** Steady state expression levels of *WRKY27*, *WRKY22* and *WRKY29* in root, shoot, leaf, flower and silique derived tissues detected by semi-quantitative RT-PCR analysis. (**B**) Relative expression of *WRKY27*, *WRKY22* and *SIRK1* derived from eFP Browser The Bio-Analytic Resource (BAR) database. **(C-G)** GUS activity was detected in transgenic P_{WRKY27}:GUS lines in total inflorescences, transmission tract of the septum, in anthers, and in the residual stigmatic papillae.

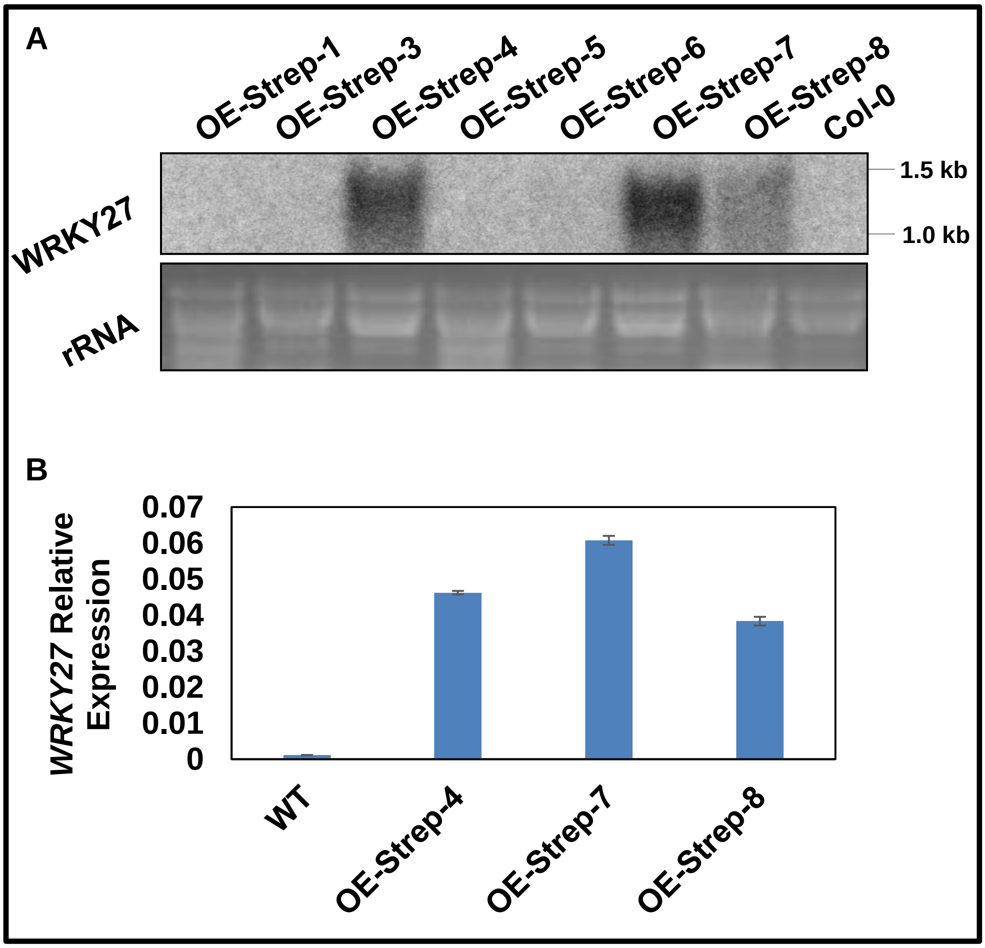

Supplementary Figure 2. Ectopic overexpression of *WRKY27*. (**A**) Northern blot analysis on eight independent *WRKY27* overexpressor *StrepII*-terminator lines. *WRKY27* transcript abundance was examined in 3 lines (*OE-Strep-4*, *OE-Strep-7* and *OE-Strep-8*). Ethidium bromide stain of rRNA was used to monitor for equal loading of the samples. (**B**) mRNA levels of *WRKY27* were detected using quantitative real-time PCR analysis in floral tissue.

Primer	sequence	Analysis
WRKY22_Forward	GTTTTGCCTGTTGAGGA	qpcr
WRKY22_Reverse	GCACTGTTCACAACCC	qpcr
WRKY27_Forward	CCGGACACATCTCCTTTATCG	qpcr
WRKY27_Reverse	CCTCTTCCTCTTCCATGTTGAC	qpcr
WRKY29_Forward	AACATACACTAATGAGCACAA	qpcr
WRKY29_Reverse	TGGTGCGTACTCGTTT	qpcr
FRK1_Forward	AAGATGGCGACTTCG	qpcr
FRK1_Reverse	GCAGGTTGGCCTGTAA	qpcr
UBQ5_Forward	GTAAACGTAGGTGAGTCC	qpcr
UBQ5_Reverse	GACGCTTCATCTCGTCC	qpcr
MS2_Forward	GTATCAGATCGCTTCTTCGG	qpcr
MS2_Reverse	TGCATGGGGATGTTTTGTA	qpcr
SPL_Forward	TCGCTAGAGCAGCTTCAGTT	qpcr
SPL_Reverse	CCTCCATTGGTCCCGTAT	qpcr
TDF1_Forward	CGGTTCCTCAAGTAGTGGG	qpcr
TDF1 _Reverse	ATGTATTCGGCTTCGATGTT	qpcr

Supplementary Table 1. Primers used to perform qRT-PCR
